# Pilot-Scale Studies of WO_3_/S-Doped g-C_3_N_4_ Heterojunction toward Photocatalytic NO_x_ Removal

**DOI:** 10.3390/ma15020633

**Published:** 2022-01-14

**Authors:** Marta Kowalkińska, Agnieszka Fiszka Borzyszkowska, Anna Grzegórska, Jakub Karczewski, Paweł Głuchowski, Marcin Łapiński, Mirosław Sawczak, Anna Zielińska-Jurek

**Affiliations:** 1 Department of Processing Engineering and Chemical Technology, Gdansk University of Technology, Gdańsk, Gabriela Narutowicza 11/12, 80-233 Gdansk, Poland; marta.kowalkinska@pg.edu.pl (M.K.); agnieszka.borzyszkowska@pg.edu.pl (A.F.B.); anna.grzegorska@pg.edu.pl (A.G.); 2 EcoTech Center, Gdańsk University of Technology, G. Narutowicza 11/12, 80-233 Gdansk, Poland; 3 Institute of Nanotechnology and Materials Engineering, Faculty of Applied Physics and Mathematics, Gdańsk University of Technology, Gabriela Narutowicza 11/12, 80-233 Gdansk, Poland; jakub.karczewski@pg.edu.pl (J.K.); marcin.lapinski@pg.edu.pl (M.Ł.); 4 Institute of Low Temperature and Structural Research, Polish Academy of Sciences, 50-422 Wroclaw, Poland; p.gluchowski@intibs.pl; 5 Centre for Plasma and Laser Engineering, The Szewalski Institute of Fluid-Flow Machinery Polish Academy of Sciences, Fiszera 14, 80-231 Gdansk, Poland; mireks@imp.gda.pl

**Keywords:** photocatalysis, NO_x_ removal, S-doped g-C_3_N_4_, WO_3_, scaling-up

## Abstract

Due to the rising concentration of toxic nitrogen oxides (NO_x_) in the air, effective methods of NO_x_ removal have been extensively studied recently. In the present study, the first developed WO_3_/S-doped g-C_3_N_4_ nanocomposite was synthesized using a facile method to remove NOx in air efficiently. The photocatalytic tests performed in a newly designed continuous-flow photoreactor with an LED array and online monitored NO_2_ and NO system allowed the investigation of photocatalyst layers at the pilot scale. The WO_3_/S-doped-g-C_3_N_4_ nanocomposite, as well as single components, were characterized by X-ray diffraction (XRD), scanning electron microscopy (SEM), Brunauer–Emmett–Teller surface area analysis (BET), X-ray fluorescence spectroscopy (XRF), X-ray photoemission spectroscopy method (XPS), UV–vis diffuse reflectance spectroscopy (DR/UV–vis), and photoluminescence spectroscopy with charge carriers’ lifetime measurements. All materials exhibited high efficiency in photocatalytic NO_2_ conversion, and 100% was reached in less than 5 min of illumination under simulated solar light. The effect of process parameters in the experimental setup together with WO_3_/S-doped g-C_3_N_4_ photocatalysts was studied in detail. Finally, the stability of the composite was tested in five subsequent cycles of photocatalytic degradation. The WO_3_/S-doped g-C_3_N_4_ was stable in time and did not undergo deactivation due to the blocking of active sites on the photocatalyst’s surface.

## 1. Introduction

The development of industry and intensified agricultural activities, energy production, and transport has led to increased NO_x_ concentration in the air and contributed to environmental degradation resulting in acid rain or fog formation. The presence of NO_x_ in the air can give rise to various toxicological responses in humans and animals, including respiratory system damage, weakening the immune system, and genetic mutation [1,2,3]. Due to harmful effects on human health, research attention is focused on controlling and decreasing the NO_x_ concentration in the air. One of the promising methods of nitrogen oxides removal is heterogeneous photocatalysis, in which the combination of semiconductor materials and light allows the generation of reactive oxygen species (ROS) to degrade emerging contaminants [4,5]. Among various methods of NO_x_ decomposition, the main advantage of photocatalytic processes is the possibility of initiating the reaction at nearly ambient temperature [2].

Over the past years, photocatalytic nitrogen oxides removal has been extensively investigated. The most described photocatalysts are based on titanium, carbon, tungsten, or bismuth compounds as a single material or a composite [1]. An example of feasible photocatalysts is graphitic carbon nitride (g-C_3_N_4_), a graphite analog and the most stable allotrope of carbon nitride at ambient conditions [6]. Graphitic carbon nitride has unique physicochemical properties such as a suitable bandgap energy (~2.7 eV), corresponding to visible-light absorption [7]. Therefore, g-C_3_N_4_ can be applied as a visible-light-driven photocatalyst toward NO_x_ removal in the gas phase, which was confirmed in the previous studies [8,9]. From the technological point of view, the synthesis of g-C_3_N_4_ is simple (typical calcination of N-containing organic precursor, e.g., urea, melamine) and low-price. However, the significant restriction of practical use of graphitic carbon nitride is a fast recombination rate of photoexcited electron-hole pairs, which lowers the photocatalytic efficiency [10]. Therefore, various modifications of g-C_3_N_4_ have been investigated, including doping to generate electron trapping centers or heterojunction in composites to enhance the charge carriers’ separation and limit the recombination process [10,11].

An effective way of improving the photocatalytic performance of g-C_3_N_4_-based material is by introducing dopants. Considering non-metal doping, incorporating sulfur with an extra electron to the crystal lattice is especially worthy of attention. According to first-principle calculations performed by Ling et al. [12], S-doping can extend the light response range by narrowing the bandgap and creating gap states. These predictions were confirmed by Kadam et al. [13], who have shown enhanced photocatalytic degradation of rhodamine B and tetracycline hydrochloride under visible light compared with an unmodified photocatalyst. Another approach to increase the photocatalytic activity is creating a heterojunction with a metal oxide such as tungsten(VI) oxide (WO_3_), an n-type semiconductor that can be activated under visible light irradiation (2.4–2.8 eV) [14].

The WO_3_/g-C_3_N_4_ composite photocatalysts in a Z-scheme heterojunction were examined towards antibiotics removal [15], dye degradation [16], and hydrogen generation [17]. Considering the promising properties of S-doped g-C_3_N_4_ compared with un-doped g-C_3_N_4_, for the first time, we propose WO_3_/S-doped g-C_3_N_4_ nanocomposite for solar-driven photodegradation of NO_x_.

**Figure 1:**
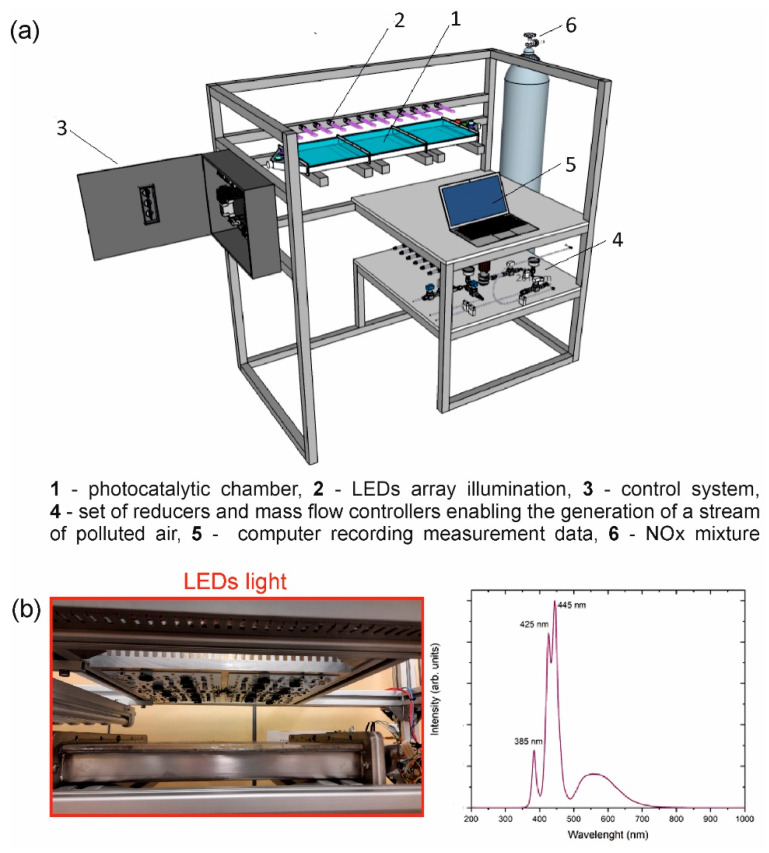
(**a**) Schematic presentation of the experimental setup of a continuous-flow photoreactor; (**b**) equipped with LED panels as irradiation source with an emission spectrum of UV–vis light.

Considering the technological point of view, we have focused on synthesizing the unsophisticated and efficient composite material. However, most of the scale-up studies and practical applications of photocatalysts considered only titania-based materials [18,19,20,21]. However, titanium(IV) oxide is efficient only under ultraviolet light [22], which is in the minority in the solar light spectrum. Therefore, more investigations in the application of visible-light-driven photocatalysts are needed. In this regard, the main goal of the present work is the pilot-scale studies of WO_3_/S-g-C_3_N_4_ toward photocatalytic NO_x_ removal in the design of a continuous-flow system. The experimental setup of a continuous-flow photoreactor is schematically presented in Figure 1. The experimental setup consists of bottles with gas mixtures of air and NO_x_, a set of reducers and mass flow controllers enabling the generation of a stream of polluted air, the photocatalytic chamber, LEDs panels 300 × 300 mm as irradiation sources, and electro-mechanical control system coupled to the computer which also secures automatic recording of measurement data and process parameters. The main element of the stand is a research chamber made of 2 mm thick stainless steel. The entire chamber consists of three modules (1) where the photocatalysts immobilized on glass plates in a dimension of 305 × 305 mm are placed. There is a free space of 5 mm above the sample for the contaminated air to flow.

At the top, the chamber is closed with a plate of quartz glass, which enables exposure of the samples to the full spectrum of simulated solar light. The light spectrum of the light source is presented in Figure 1b. The light flux in the range of 310–380 nm was 5 mW·cm^−2^. The air is supplied to the chamber using mass flow controllers (Bronkhorst F-201CV-10K-RGD-00-V), enabling digital flow control in the range of 0.2–10 nL/min (with an accuracy of ±0.5% of reading plus ±0.1% range). The connected chambers on both sides end with diffusers (2), ensuring even gas distribution. Gas chemical sensors (3) are installed in the diffusers, enabling the measurement of the following parameters: the 4-electrode electrochemical sensor for nitrogen oxide concentration measurement, the 4-electrode electrochemical sensor for nitrogen dioxide concentration measurement, temperature, humidity, and pressure sensors.

The degradation mechanism and effect of process parameters (humidity, gas flow rate, NO_2_ concentration, and amount of photocatalyst) on NO_x_ removal efficiency were studied in detail.

## 2. Materials and Methods

### 2.1. Materials

Melamine (ACS reagent, 99.0–100.5%) and thiourea (ACS reagent, ≥99%) for the synthesis of S-doped-g-C_3_N_4_, sodium tungstate dihydrate (ACS reagent, ≥99%), and oxalic acid (98%) for the synthesis of WO_3_ were purchased from Sigma-Aldrich (Poznan, Poland). Ethanol (96%) for WO_3_/S-doped-g-C_3_N_4_ synthesis was purchased from POCh. All reagents were used as received without further purification. Deionized water (DI) was used in all experiments.

### 2.2. Synthesis of Photocatalysts

#### 2.2.1. Synthesis of WO_3_

Firstly, 7.34 g of Na_2_WO_4_ 2H_2_O was dissolved in 90 cm^3^ of DI water and 2M HCl with the volume ratio of 1:1 and marked as solution A. Next, 0.35 g of oxalic acid was dissolved in 90 cm^3^ of DI water and marked as solution B. Solution A was added to solution B and mixed for 15 min. The mixture was transferred to a 200 cm^3^ Teflon-lined stainless steel reactor, and solvothermal synthesis was performed at 90 °C for 3 h. The obtained material was rinsed three times with DI water, once with methanol, and then dried at 80 °C for 2 h. Finally, the powder was calcined at 500 °C for 1 h with a heating rate of 5 °C/min.

#### 2.2.2. Synthesis of S-Doped-g-C_3_N_4_

In the first step, 7.5 g of melamine and 7.5 g of thiourea were ground together in a mortar. Then, the material was calcined at 580 °C for 3 h with a heating rate of 10 °C/min.

#### 2.2.3. Synthesis of WO_3_/S-Doped-g-C_3_N_4_ Composite

Typically, 3 g of S-doped-g-C_3_N_4_ was dispersed in a 100 cm^3^ mixture of DI water and ethanol with the volume ratio of 1:1 and ultrasonicated for 1 h. Then, 3 g of WO_3_ was added and ultrasonicated for another 1 h. The resulting suspension was transferred to a 200 cm^3^ Teflon-lined stainless steel reactor, and solvothermal synthesis was performed at 150 °C for 6 h with a heating rate of 5 °C/min. The obtained material was rinsed a few times with DI water and dried at 80 °C for 3 h.

### 2.3. Material Characterization

The structure and phase composition of synthesized photocatalysts were investigated using X-ray powder diffraction (XRD). X-ray diffraction patterns were recorded on the Rigaku MiniFlex 600 X-ray diffractometer (Tokyo, Japan) with Cu Kɑ radiation (λ~1.54 Å) in the 2*θ* = 10–80° range. Moreover, the chemical structure and bond identification in samples was determined by Fourier-transform infrared spectroscopy (FTIR) in the transmittance mode. The FTIR Nicolet iS10 (Thermo Fisher Scientific, Waltham, MA, USA) spectrometer was used at room temperature in the wavenumber range from 4000 to 400 cm^−1^. The pellets containing 95% of potassium bromide and 5% (wt.) of a photocatalyst were analyzed in each measurement.

The morphologies of prepared powders were characterized by scanning electron microscope (SEM) with field emission gun, model Quanta FEG 250 (FEI, Hillsboro, OR, USA). The content of sulfur and WO_3_ in the composite were measured using a WDXRF S8 TIGER analyzer (Bruker, S8 TIGER, Bruker, Karlsruhe, Germany) using X-ray fluorescence spectroscopy (XRF). The analyses were performed according to the standard PN-EN 15309:2010. To determine the parameters of the porous structure of the single components and the composite, including Brunauer–Emmett–Teller (BET) surface area and pore volume using low-temperature nitrogen sorption, a Micromeritics Gemini V apparatus (model 2365; Norcross, GA, USA) was used. Before each measurement, the analyzed photocatalysts were degassed at 200 °C for 2 h. The specific surface areas were determined by the multipoint BET method in the *p/p*_0_ range from 0.05 to 0.30. Total pore volumes were estimated from the nitrogen adsorbed at *p/p*_0_ = 0.995.

The detailed chemical composition of the samples and nitrates identification were analyzed by the X-ray photoemission spectroscopy method (XPS). XPS measurements were carried out at room temperature under ultrahigh vacuum conditions with pressures below 1.1 × 10^−6^ Pa using Omicron NanoScience equipment with an Argus hemispherical analyzer (ScientaOmicron, Uppsala, Sweden). Spectra deconvolution was performed with the CASA XPS software (Casa Software Ltd., ver. 2.3.23., Devon, UK) package using the Shirley background subtraction and Gauss–Lorentz curve fitting algorithm by the least-squares method—GL (30). The XPS spectra were calibrated to obtain binding energy of 285.00 eV for the C 1 s peak.

The optical properties, including absorbance and Kubelka–Munk functions, were studied by a UV–vis spectrophotometer (Thermo Fisher Scientific Evolution 220, Waltham, MA, USA) to measure diffuse reflectance (DRS). During measurements, barium sulfate was used as a standard. Based on the obtained spectra, the bandgap energy calculations were performed by Tauc’s method.

The photoluminescence spectra were recorded using an FLS980 fluorescence spectrophotometer from Edinburg Instruments (Livingston, Wielka Brytania) equipped with a 450 W xenon lamp as an excitation source and a Hamamatsu 928 PMT detector. Measured PL spectra were corrected for the sensitivity and wavelength of the experimental setup. The slit width was 2 μm (excitation spectra) or 0.3 μm (emission spectra). The same equipment was used for decay measurements to calculate the charge carrier lifetime, and the lamp was changed to a 150 W pulse xenon lamp. All measurements were recorded at room temperature. The average experimental lifetimes of obtained photocatalysts were calculated according to Hu et al. [23].

### 2.4. Coating Methodology

Firstly, 2 g of the photocatalyst was mixed with deionized water (30 cm^3^) in Falcon tubes (50 cm^3^) using the vortex mixer. The homogenous sample was transferred onto glass (30.5 cm × 30.5 cm) and evenly distributed on the whole surface. Finally, the coatings were dried at room temperature for 24 h.

### 2.5. Photocatalytic Setup

The photocatalytic experiments were carried out in a flat quartz glass photoreactor in a volume of 1.5 dm^3^ with continuous NO_2_ flow. The photoreactor was used for NO_2_ photocatalytic conversion over WO_3_, S-doped g-C_3_N_4,_ and WO_3_/S-doped g-C_3_N_4_, as depicted in Figure 1a. The plate with the photocatalyst is parallel to an air-tight optical window for photoirradiation. The gas with pollution passes only through the space between the test plate and the window. The flow rates of the reactant gases (100 ppm NO_2_ in synthetic air, Linde Gas) and the pretreatment gas (synthetic air, Linde Gas) were adjusted with gas flow meters. The relative humidity (RH) level of the NO_2_ flow was controlled at 40% by passing the zero air stream through a humidification chamber. The air stream, NO_2_, and humidity flow rates were controlled at 0.9 dm^3^/min, 0.1 dm^3^/min, and 0.3 dm^3^/min. The concentration of NO_2_ was detected continuously by employing a NO_x_ analyzer (AlphaSense, NO-B4 4-electrode nitric oxide sensor, Alphasense 000-0NO2-B43F nitric dioxide sensor). A LED panel light was used in the photocatalytic reaction as a light source. The light flux in the range of 310–380 nm was on average 5 mW·cm^−2^.

Before each measurement, the test gas was allowed to flow into the photoreactor without photoirradiation to reach adsorption-desorption equilibrium. After 30 min, the lamp was turned on to start the photocatalytic process. Finally, the photocatalytic performance of obtained photocatalysts NO_2_ was demonstrated in terms of NO_2_ conversion rate (Equation (1)):

(1) $$NO_{2}\;conversion=\left(\frac{\left[NO_{2\;inlet}\right]-\left[NO_{2\;outlet}\right]}{\left[NO_{2\;inlet}\right]}\right)\times 100\%.$$

## 3. Results and Discussion

### 3.1. Characterization of Photocatalysts

**Figure 2:**
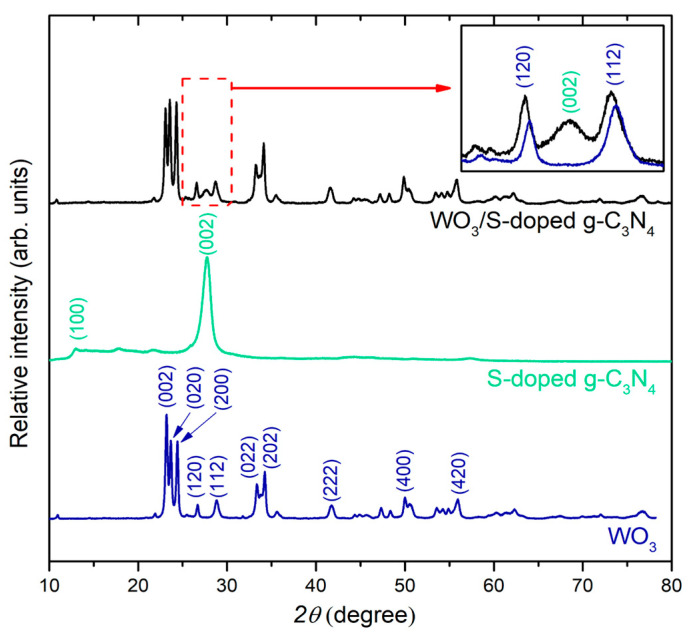
XRD patterns of WO_3_/S-g-C_3_N_4_ and single components. The inset shows the zoom at diffractograms of WO_3_ and WO_3_/S-g-C_3_N_4_ in the range 2*θ* = 25°–30°.

The structural parameters and phase compositions of photocatalysts were examined using X-ray diffraction. XRD patterns are presented in Figure 2. The XRD patterns of the single-phase components monoclinic WO_3_ and S-g-C_3_N_4_ are in good agreement between the recorded and calculated patterns. No signals originating from impurities were detected. The diffractogram of WO_3_/S-g-C_3_N_4_ was similar to pure tungsten(VI) oxide patterns. However, a significant difference is noted in the range of 2*θ* = 25°–30° (inset in Figure 2). In the presented composite, the peaks (120) and (112) originating from monoclinic WO_3_ and the main peak (002) from S-g-C_3_N_4_ can be distinguished. This analysis confirmed the presence of a hybrid photocatalyst of graphitic carbon nitride and WO_3_.

**Figure 3:**
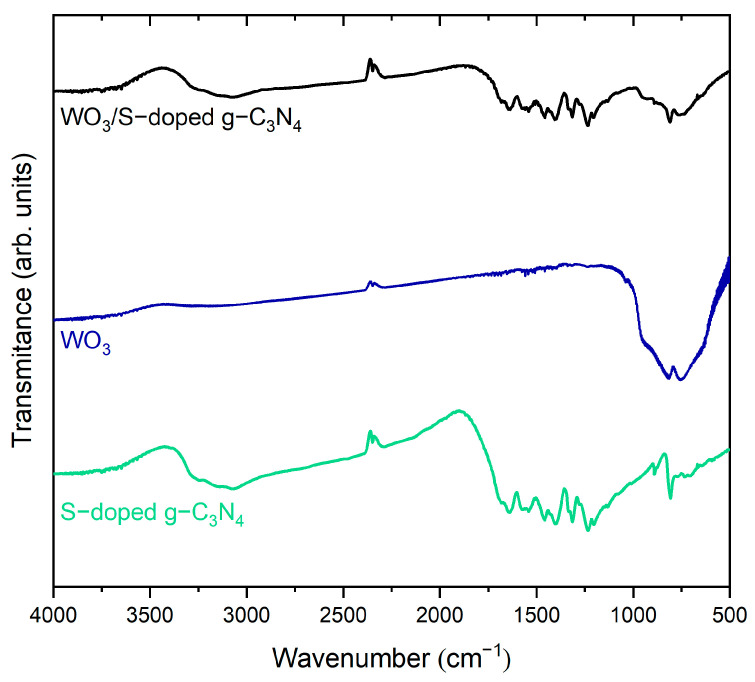
FTIR spectra of WO_3_/S-g-C_3_N_4_ and single components.

The chemical bonding identification of obtained samples was investigated by infrared spectroscopy. The presence of characteristic bands in single components of WO_3_ and S-g-C_3_N_4_ confirmed the previous phase identification of WO_3_/S-g-C_3_N_4_ (see Figure 3). In the case of pure WO_3_, a broad absorption band in the wavenumber range of 420–1000 cm^−1^ attributed to the vibration modes of the W–O bond verifies the formation of tungsten(VI) oxide. The strong bands in 745 cm^−1^ and 818 cm^−1^ are assigned to the stretching mode of W-O-W [24]. These peaks probably overlap with a band located at 946 cm^−1^, attributed to W=O stretching vibration [25,26]. In the case of S-doped graphitic carbon nitride, a narrow band at 808 cm^−1^ can be attributed to the triazine ring mode, which corresponds to condensed CN heterocycles [27,28]. The distinct absorption bands at 1572 and 1639 cm^−1^ are attributed to C=N stretching, while the four bands at 1231, 1314, 1396, and 1453 cm^−1^ to aromatic C–N stretching [29]. In the composite, FTIR spectra are similar to the doped carbon nitride. The difference can be noticed below 1000 cm^−1^, in which triazine ring modes from S-g-C_3_N_4_ overlap with bands assigned to the stretching mode of W–O–W. Therefore, FTIR spectra confirmed the presence of two phases in the WO_3_/S-g-C_3_N_4_ composite.

**Figure 4:**
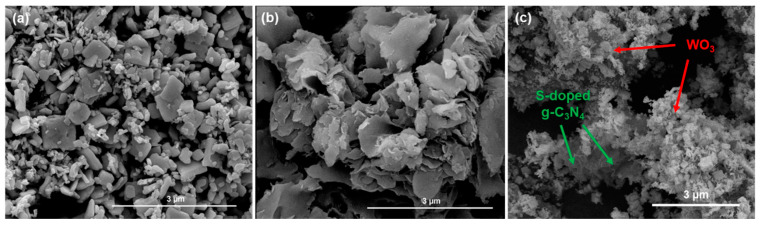
SEM images of (**a**) WO_3_; (**b**) S-g-C_3_N_4_; and (**c**) WO_3_/S-g-C_3_N_4_.

The morphology of the WO_3_/S-g-C_3_N_4_ composite and single components was determined by scanning electron microscopy. SEM images of the photocatalysts are presented in Figure 4. WO_3_ possessed a polydisperse nature in which small nanoparticles and disc-shaped nanostructures can be distinguished (Figure 4a). In the case of S-g-C_3_N_4_ (Figure 4b), a flake-like morphology was observed, which is in agreement with previous reports [6,7,13]. Comparing SEM images of the composite (Figure 4c) with the morphology of single components, features of two phases can be indicated. The two-dimensional flakes of S-g-C_3_N_4_ are covered with nanoparticles of WO_3_. Therefore, each phase is connected with the other, which results in creating a heterojunction.

Moreover, the surface area of obtained samples was calculated using the BET method. The values of specific surface areas and pore volumes are presented in Table 1. All materials possessed surface area in the range of 11.5–14.5 m^2^/g. Moreover, to confirm the presence of sulfur in the WO_3_/S-g-C_3_N_4_ composite, X-ray fluorescence spectroscopy (XRF) was performed. The content of S in the binary WO_3_/S-g-C_3_N_4_ (1:1) photocatalyst was about 0.03–0.05% (m/m). Lin et al. [30] have shown that the high dopant concentration can hinder photocatalytic activity. In the case of Fe-doped TiO_2_, it was demonstrated that the extent of photodegradation diminishes with increasing dopant concentration, and only the 0.01 mol% Fe-TiO_2_ film showed enhanced photoactivity. It resulted from the presence of lattice distortions at high dopant amounts, which increased the number of recombination centers, hindering photocatalytic activity [25].

**Figure 5:**
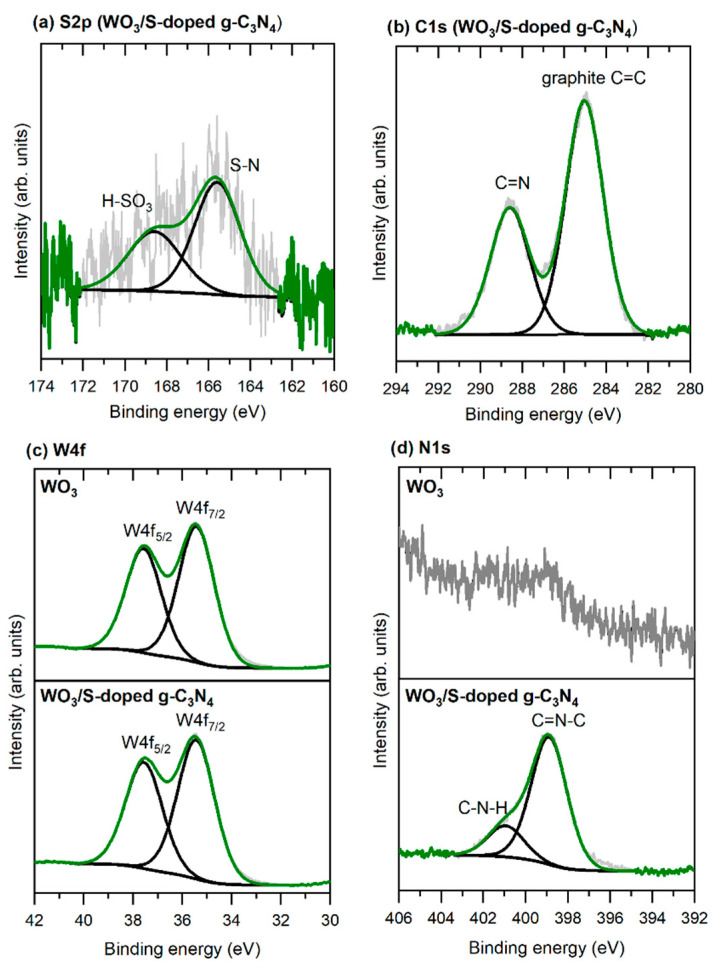
XPS spectra of (**a**) S2p, (**b**) C1s, (**c**) W4f, and (**d**) N1s signals for WO_3_ and WO_3_/S-doped g-C_3_N_4_.

To study the chemical composition of the surface WO_3_ and WO_3_/S-doped g-C_3_N_4_ photocatalysts after the photocatalytic NO_x_ conversion, high-resolution X-ray photoelectron spectroscopy (XPS) analyses were performed. Further analysis of the final composite material confirmed the presence of sulfur (Figure 5a). The peaks at 165.5 eV and 168.5 eV originate from N–S groups and H–SO_3_ groups present in the S-doped carbon nitride [31]. On the basis of survey spectra, it can be concluded that the sulfur content is relatively low compared with other elements, which is in agreement with XRF results. In the case of XPS spectra of the C1s regions (Figure 5b), the experimental curve can be deconvoluted into two peaks with binding energies at 285 eV and 288.5 eV, which could be ascribed to C–C and/or graphite C=C surface coordination of carbon, and sp^2^-bonded carbon (C=N), respectively, which are typical groups for g-C_3_N_4_ [32,33]. The doublet peaks in Figure 5c, located at a binding energy of 35.5 eV and 37.6 eV, are attributed to W4f_7/2_ (W^+5^) and W4f_5/2_(W^+6^), respectively [14]. No sub-stoichiometry of tungsten(VI) oxide and the WO_3_/S-g-C_3_N_4_ was observed. The N1s signal (Figure 5d) may be deconvoluted to two peaks at 399 eV and 401 eV, which corresponds to triazine rings (C=N–C) and C–N–H, respectively [32,34,35]. For pure WO_3_ N1s region weak broad peak about 400 eV may be assigned to the unreacted NO adsorbed on the surface [36].

**Figure 6:**
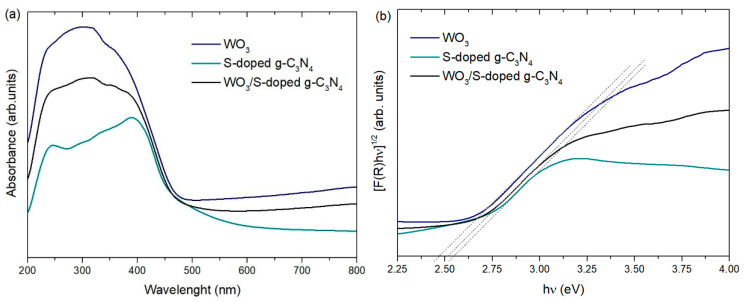
(**a**) DR/UV–vis spectra and (**b**) the Kubelka–Munk transformation of obtained samples.

The DR/UV–vis spectra of WO_3_, S-doped g-C_3_N_4_, and WO_3_/S-g-C_3_N_4_ are presented in Figure 6. All materials revealed the highest absorbance in the range from 200–450 nm. These photocatalysts are also capable of absorbing visible light at higher wavelengths, which is consistent with the fact that all samples are colorful (WO_3_ is lime-green, whereas S-doped g-C_3_N_4_ is yellow, which corresponds to light absorption in the 530–590 nm range). Based on this observation and the calculated bandgap values, it was assumed that all samples could efficiently utilize visible light, which is in the majority of the solar light spectrum.

**Figure 7:**
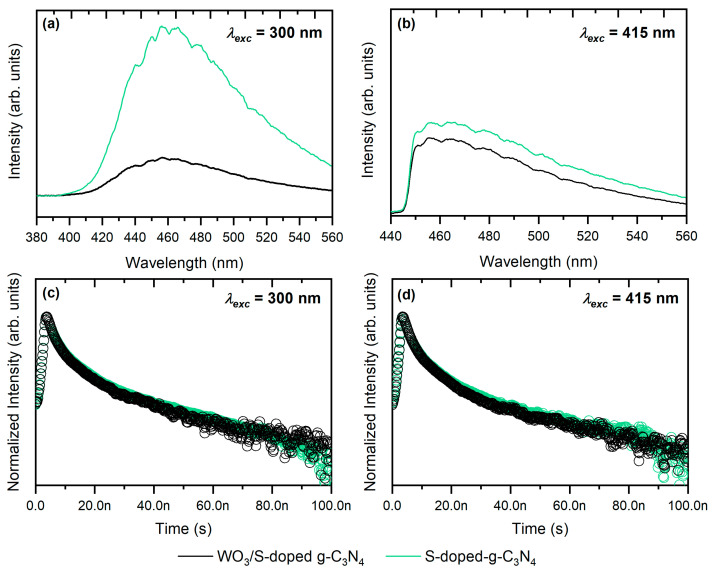
(**a**,**b**) Photoluminescence spectra and (**c**,**d**) luminescence decay curves of S-doped g-C_3_N_4_ and WO_3_/S-doped g-C_3_N_4_ composite under different excitation wavelengths.

Moreover, photoluminescence spectra and luminescence decay curves were determined, as presented in Figure 7. The highest PL emission was observed for S-doped g-C_3_N_4_ with the maximum at ~455 nm (blue light) under UV-light excitation (Figure 7a). Similar observations were noticed when the sample was excited in visible light (Figure 7b). According to Zhang et al. [7], carbon nitride can be characterized by enhanced photoresponse and an extremely high quantum yield up to 19.6%. Although these properties are attractive in many potential applications, e.g., bioimaging, in the case of photocatalysis, the high PL emission may hinder the photocatalytic activity. The emission is connected with radiative recombination, which is not desirable because this process lowers the charge carriers’ concentration [4]. Therefore, the photocatalytic processes with g-C_3_N_4_ as a single compound are insufficient and require further photocatalyst modification [37]. The lower PL intensity for WO_3_/S-g-C_3_N_4_ in comparison with pure S-doped carbon nitride was noticed. The most visible differences are especially in Figure 6a under *λ_exc_* = 300 nm conditions. These results confirm the limited recombination rate due to efficient charge carriers’ separation in the heterojunction [38]. Furthermore, charge carriers’ lifetimes were calculated based on luminescence decay curves (Figure 7c,d). The average carrier lifetimes *<τ>* are presented in Table 1. All photocatalysts possessed the *<τ>* value of 14–16 ns. It is worth highlighting that the charge carrier lifetimes do not decrease when the light excitation wavelength is changed from 300 nm (UV) to 415 nm (visible). This is another confirmation that these materials are photoactive under solar irradiation. Moreover, there is no significant decline in the *<τ>* value when S-doped carbon nitride is introduced into the composite with WO_3_. Under 300 nm excitation conditions, the carrier lifetime is prolonged in comparison with single S-g-C_3_N_4_, which can be explained by efficient charge carriers’ separation. This process resulted in enhanced photocatalytic performance.

### 3.2. Photocatalytic Activity and Degradation Mechanism

**Figure 8:**
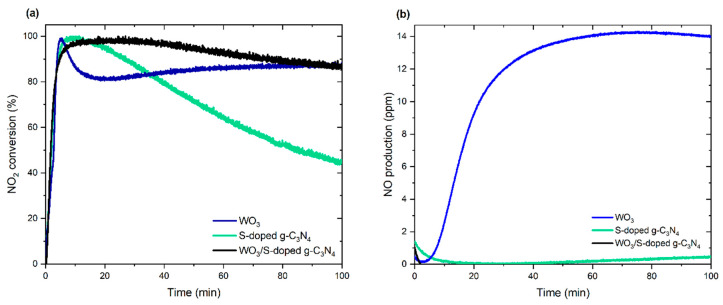
(**a**) Photocatalytic NO_2_ conversion and (**b**) NO production using obtained photocatalysts.

The photocatalytic activity of the WO_3_/S-g-C_3_N_4_ composite, as well as single-phase photocatalysts, were demonstrated towards NO_2_ conversion in a continuous-flow system under simulated solar light irradiation. The initial concentration of NO_2_ was about 5 ppm. As shown in Figure 8a, WO_3_/S-doped g-C_3_N_4_ composite exhibited near 100% NO_2_ removal within 5 min of the process. The high efficiency of this composite is almost constant within time. In the case of pure WO_3_ and S-doped g-C_3_N_4,_ rapid removal of NO_2_ concentration was also observed; however, photocatalytic activity decreased within the time, which was particularly noticeable for S-doped g-C_3_N_4_. S-doped g-C_3_N_4_ was supposed to transform NO_2_ to forms, which caused blocking of the active sites on the photocatalyst’s surface [39]. These results were compared with NO production (Figure 8b). Noticeably, the NO_2_ → NO conversion is favorable for the WO_3_ single compound. Che-Chin Yu et al. [40] reported the phototransformation of NO_2_ into NO in the N_2_ presence over WO_3_ photocatalyst regarding the following reaction: NO_2_ + N_2_ → 2NO. It is consistent with the probable presence of unreacted NO adsorbed on the surface, indicated by XPS spectra. Therefore, reduction processes are predominant in the case of tungsten(VI) oxide.

Considering the overall degradation of NO_x_ species, only the composite WO_3_/S-doped g-C_3_N_4_ allowed the complete degradation of NO_2_ and did not cause the production of NO. According to the available literature reports, two possible explanations of this observation for the composite are possible. First, the surface of the binary composite is more reactive than for single components because of the more efficient charge carriers’ separation. A similar effect was described by Méndez-Román and Cardona-Martínez [41], who investigated SiO_2_–TiO_2_ photocatalysts towards oxidation of toluene. The composite exhibited better photocatalytic performance and lower deactivation than TiO_2_ due to higher toluene adsorption capacity and changes in Brønsted acidity of intermediates. Therefore, new surface sites would be responsible for the enhanced photocatalytic performance. The second possibility is a change of photocatalytic mechanism when WO_3_ and S-doped g-C_3_N_4_ are present in the heterojunction. However, changes in charge carriers’ transport in the composite caused the promotion of NO_2_ reduction to gaseous NO and N_2_. In this case, less NO_3_^−^ was adsorbed on the photocatalyst surface than from NO_2_ oxidation [42]. Therefore, according to Dalton et al. [36], XPS spectroscopy is needed as a technique suitable for surface analysis. However, the accurate indication of the NO_2_ conversion pathway is difficult for WO_3_/S-doped g-C_3_N_4_ using XPS spectroscopy because of the predominant C-N signals in N1s spectra. This inconvenience was described precisely by Li et al. [43], who modified graphitic carbon nitride using nitric acid or ammonia gas atmosphere. Remarkably, there were no differences in XPS spectra of N1s signals, although the adsorption of nitrates or ammonia was certain. In this study, the negligible NO production during the photocatalytic process in the presence of composite photocatalyst may result from advanced photodecomposition without NO as a main intermediate product. The second possibility is photooxidation to nitrates. However, in this case, blocking active sites and a further decrease in activity should be observed, whereas the performed subsequent degradation cycles showed the long-term stability of the WO_3_/S-doped g-C_3_N_4_ composite material [44,45].

#### 3.2.1. Effect of Process Parameters

**Figure 9:**
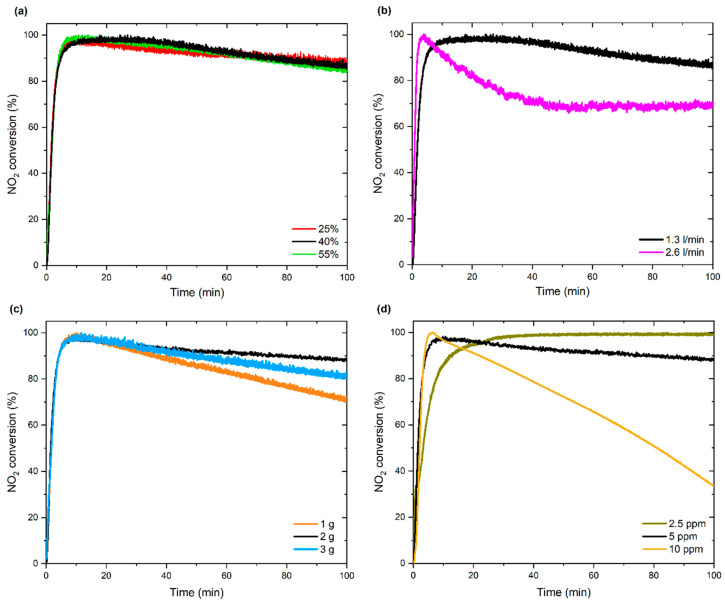
Effect of: (**a**) humidity, (**b**) gas flow, (**c**) photocatalyst amount, and (**d**) NO_2_ initial concentration on NO_2_ conversion using WO_3_/S-doped g-C_3_N_4_.

The photocatalytic process, including NO_2_ removal, depends on different factors, including temperature, relative humidity, pollutant concentration, and the amount of photocatalyst [31,32]. Therefore, in this study, the effect of these parameters on photocatalytic activity was evaluated. Three relative humidity values were set as 25%, 40%, and 55%, respectively. As shown in Figure 9a for the WO_3_/S-doped g-C_3_N_4_ composite, relative humidity had no significant effect on the NO_2_ conversion. Other observations were reported by Maggos et al. [46] during photocatalytic NO_x_ removal in the presence of TiO_2_. In their study, humidity plays a crucial role in the photooxidation process since water contributes to reactive oxygen species (ROS) formation. From the technological point of view, eliminating the humidity effect can allow photocatalysis without the dependence on external weather conditions.

Afterward, the effect of gas flow rate was evaluated and fixed on 1.3 dm^3^/min and 2.6 dm^3^/min. In contrast to observations from the influence of humidity, the gas flow rate was a significant parameter affecting the photocatalytic efficiency, as shown in Figure 9b. A two-fold increase in gas flow rate caused a remarkable decrease in NO_2_ conversion. This may be related to the short contact time between pollutant and photocatalyst surface during the passage of higher gas flow rate [47]. A similar effect was observed by Li et al. [48] in photocatalytic depolymerization of the oxidized β-O-4 molecule in the continuous-flow reactor. The authors noticed that too fast flow rate reduced photocatalytic efficiency due to the short contact time between the reactant and photocatalyst, while when the flow rate was too slow, performance was limited due to the possible mass transfer resistance.

Subsequently, the effect of photocatalyst dose was investigated (Figure 9c). Firstly, the chosen amount of photocatalyst—2 g for the glass plate in the size 30.5 cm × 30.5 cm—allowed for the highest conversion of NO_2_. Other investigated doses: 1 g and 3 g caused the depletion of NO_2_ removal within the experimental time. The observed reduction of NO_2_ conversion during the process with the smaller amount of the photocatalyst could be explained by the smaller presence of available active sites. On the other hand, the rise of the catalyst dose could cause the covering of some photocatalysts particles, thereby cutting off the radiation access.

Finally, the NO_2_ initial concentration effect on NO_2_ conversion was examined. The selected initial concentrations of NO_2_ were as follows: 2.5 ppm, 5.0 ppm, and 10.0 ppm. As shown in Figure 9d, 100% of NO_2_ conversion was maintained after 5 min and 30 min, respectively, for 5 ppm and 2.5 ppm. On the contrary, during the process with the initial concentration of 10 ppm, the effectiveness of NO_2_ removal decreased over time from 100% after 5 min up to 37% after 100 min.

#### 3.2.2. Stability Tests

**Figure 10:**
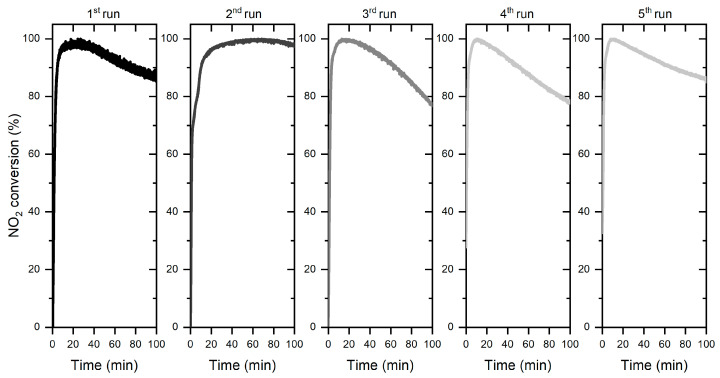
Cycling test of the photocatalytic NO_2_ removal using WO_3_/S-doped g-C_3_N_4_.

The stability of the WO_3_/S-doped g-C_3_N_4_ composite was evaluated in five subsequent cycles, as presented in Figure 10. The decline of NO_2_ conversion to ca. 80% was observed. This is consistent with the fact that adsorption of the NO_x_ and water molecules are necessary to initiate the photocatalytic process, which can block the active sites. However, the second and fifth cycles with increased photocatalytic efficiency confirmed that the WO_3_/S-doped g-C_3_N_4_ material is capable of desorbing the adsorbed species and unblocking the active species for better NO_2_ conversion. This observation confirms that the obtained composite showed long-term stability. 

**Figure 11:**
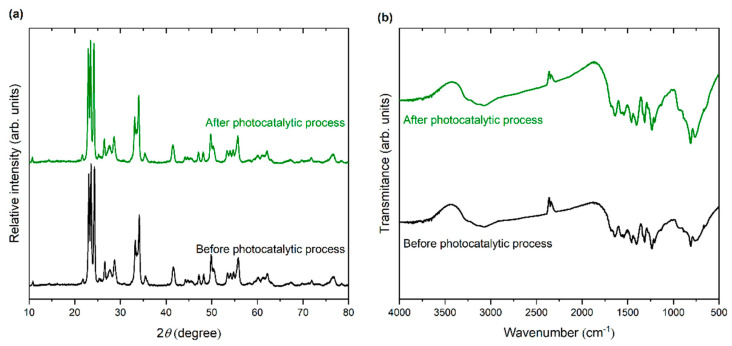
(**a**) XRD patterns and (**b**) FTIR spectra of WO_3_/S-doped g-C_3_N_4_ before and after the photocatalytic process.

In addition, XRD analysis and FTIR spectra after the photocatalytic process were performed. As presented in Figure 11a, no signals on XRD patterns, which can originate from the presence of an additional phase, were noticed. Similar conclusions can be noted from FTIR spectra in Figure 11b; no additional bands were detected after the photocatalytic process.

## 4. Conclusions

In this study, the photocatalytic performance of WO_3_/S-doped g-C_3_N_4_ nanocomposite towards NO_x_ removal was studied for the first time. The experiment was performed in a continuous-flow photoreactor on the pilot scale. Simulated solar light emitted by a newly designed LED array was used as a light source. The highly efficient composites were successfully obtained without additional phases, confirmed by X-ray diffraction. SEM images of the nanomaterials showed the nanocomposite morphology as two-dimensional. S-doped g-C_3_N_4_ nanosheets were covered by WO_3_ nanoparticles. XPS and XRF spectroscopy confirmed the presence of sulfur in the final photocatalyst. High absorbance and suitable bandgap value proved that the obtained photocatalysts utilize solar light efficiently. The photoluminescence spectra and carriers’ lifetime calculations showed better separation of charge carriers in the composite than in a single component. Finally, WO_3_, S-doped g-C_3_N_4,_ and WO_3_/S-doped g-C_3_N_4_ were characterized by fast photocatalytic NO_2_ removal. Almost 100% of NO_2_ was degraded in less than 5 min of the process. For S-doped g-C_3_N_4_ nanosheets and WO_3_ nanoparticles, active sites were further blocked, which caused a decrease in NO_2_ removal. This effect was not observed for the WO_3_/S-doped g-C_3_N_4_ composite. Additionally, the newly developed composite efficiently degraded NO_2_ without the simultaneous production of NO. Remarkably, the composite photocatalyst was tested in the subsequent degradation cycles, which confirmed the long-term stability of the composite. No structural changes were noticed after the photocatalytic processes.

To summarize, the pilot-scale studies of the photocatalysts towards NO_x_ conversion under simulated solar light were demonstrated. The high photocatalytic activity of WO_3_/S-doped g-C_3_N_4_ and simple photocatalyst synthesis encourages the industrial application of the obtained nanocomposites in air purification processes.

## Figures and Tables

**Table 1:** Morphological and optical properties of obtained samples.

Sample	BET Surface Area (m^2^/g)	Pore Volume (cm^3^/g)	Bandgap (eV)	Average Carrier Lifetime (ns)	
				300 nm	415 nm
WO_3_	14.0	0.0069	2.48	-	-
S-doped g-C_3_N_4_	14.5	0.0073	2.55	15.3	16.1
WO_3_/S-doped g-C_3_N_4_	11.5	0.0058	2.51	17.6	14.2

## Data Availability

The authors confirm that the data supporting the findings of this study are available within the article. Derived data supporting the findings of this study are available from the corresponding author [AZJ] on request.
